# Evaluation of parental stress in neonatal intensive care unit in Iran: a national study

**DOI:** 10.1186/s12912-023-01200-4

**Published:** 2023-02-15

**Authors:** Mohammad Heidarzadeh, Haydeh Heidari, Ali Ahmadi, Kamal Solati, Narges sadeghi

**Affiliations:** 1 Zahedan University of Medical Sciences, Zahedan, Iran; 2 Faculty of Nursing and Midwifery, Modeling in Health Research Center, Shahrekord University of Medical Sciences, Shahrekord, Iran; 3 Department of Epidemiology and Biostatistics, School of Health and Modeling in Health Research Center, Shahrekord University of Medical Sciences, Shahrekord, Iran; 4 Modeling in Health Research Center, Shahrekord University of Medical Sciences, Shahrekord, Iran; 5 Islamic Azad University, Isfahan, Iran

**Keywords:** Parents, Stress, Neonatal Intensive Care Unit, Health Care System

## Abstract

**Background:**

More attention is paid to the survival and treatment of the sick infant in the neonatal intensive care unit (NICU) and parental stress is not considered. The purpose of this study was to determine samples of the level of parental stress in the NICU.

**Method:**

This study is a descriptive-analytical study in which Parental Stress and General Health were used in an analytical national survey in Iran. The research sample consists of 2456 parents of infants admitted to NICU. The sampling method was multi-stage random. We used 11- item parental stress questionnaire and 28-item general health questionnaire for the data collection.

**Result:**

Baloch ethnicity with an average of 11.52 had the highest level of stress. The mean stress score of mothers was higher than fathers. The mean score of all dimensions of physical symptoms, anxiety, social functioning, depression, and total mental health score in mothers was higher than fathers. There was a statistically significant difference in the length of hospitalization in terms of different levels of parental stress scores (*p* < 0.002). Lack of decisive response to parents was one of the most stressful issues (8.1%).

**Conclusion:**

Our result shows mothers' stress was higher than fathers. So that health policymakers should pay attention to stress risk factors to provide appropriate interventions according stress risk factors Future studies should design appropriate interventions to reduce parental stress, especially in high-risk mothers.

## Introduction

The environment of the neonatal intensive care unit (NICU) is full of stressful stimuli due to complex technology and numerous treatment procedures [1]. When an infant is admitted to the NICU, the process of parental attachment is disrupted due to the special conditions of this unit. Also, the parents are worried about the infant's health and are concerned about the situation of the whole family [2]. To reduce parental stress, educational programs, parent empowerment interventions, and behavioral and psychological strategies and interventions are of particular importance [3]. The use of appropriate interventions and strategies increases parental self-efficacy and promotes physical and emotional communication between parent and child [4]. One of the ways to support the parents and reduce their stress is family-centered care; nurses have a key role in providing it successfully [5]. Parental support promotes proper parent–child interaction and improves the attachment process [6].

Approximately 15 million premature infants are born worldwide each year [7]. Prematurity rates are estimated at 5.6 to 39.4% in Iran [8]. Parents of a hospitalized infant may spend weeks or months in the NICU [9]. Parents suffer from various psychological reactions following the infant's hospitalization in the NICU [10, 11]. Hospitalized infant mothers feel very tired; unable to care for the infant due to critical conditions and complex equipment connected to the infant [12]. These long-term consequences change family functioning and the mental health of parents [13, 14].

Parental stress is a major problem worldwide due to the admission of a infant in the NICU [15]. Parental stress begins when the infant is admitted to NICU, although some parents experience depression and post-traumatic stress up to 1 year after NICU discharge [16]. More attention is paid to the survival and treatment of the sick infant in the NICU and parental stress is not considered [17]. The results of studies on parental stress vary in the NICU [18–20]. The results of a study showed that fathers experience more stress [18]. Researchers showed that mothers experience more stress in the NICU due to changing parental roles [19, 20]. The results of another study showed that mothers and fathers experience the same stress in the NICU [21].Previous studies stated that in order to provide appropriate interventions to improve the health of parents, it is necessary to determine the risk factors related to parental stress [22, 23]. Several studies stated that due to differences in NICU, the factors related to parental stress are different, so the health care team should focus more attention on recognizing these factors [14, 24]. The above studies showed various contradictions in the field of parental stress. In most of them, the dimensions of stress were examined and the present study is the first study that uses a questionnaire to determine the level of parental stress on a large neonatal intensive care unit. Therefore, the specific purposes of this study were to answer following questions:

- What is the level of parental stress?
- What is the level mental health of parents?
- Are the levels of stress and mental health different between fathers and mothers?

## Methods

This study is a descriptive-analytical study in which the Parental Stress and General Health were used in an analytical survey that aimed to determine the mental health status of parents in the NICU in the form of a national study.

### Samples

The research sample consists of 2500 parents of infants admitted to the NICU who were selected from the research community in several stages.

The standard deviation was 1.6 and α = 0.05 (95% confidence interval and z = 1.96) was considered.

$$n=\frac{{z}^{2}\cdot p\left(1-p\right) }{{d}^{2}}= \frac{{\left(1/96\right)}^{2} \times 0/7\times 0/3}{{\left(0/05\right)}^{2}}=2500$$

Two thousand seven hundred questionnaires were distributed and 2456 questionnaires were received.

The sampling method was multi-stage random (more than one method). First, the whole country was divided into 10 health zones of the Ministry of Health. In the next stage, the stratified sampling was used. Our stratum was in the health zone. The number of hospital beds was considered as the sampling weight, the statistics of which were extracted from the Health Department of the Ministry of Health and Medical Education. The number of samples in each hospital was selected in proportion to the number of NICU beds. After that, in each hospital, sampling was done by census manner of parents of infants admitted to the NICU who met the inclusion criteria, with informed consent. The sampling period lasted 8 months. Inclusion criteria included parents with infants hospitalized in the NICU, 24 h after hospitalization of the infant, and willingness to participate in the study. Exclusion criteria were unwillingness to continue participating in the study.

### Data collection

First, research approval and a code of ethics were obtained. Before starting the executive stages of the research, a one-day briefing session was held to become familiar with the plan and to understand how to complete the questionnaires and informed consent form with the neonatal health experts in one room of the Ministry of Health and Medical Education. In cooperation with the Ministry of Health, questionnaires and informed consent forms were provided to neonatal health experts.

Questionnaires were distributed throughout the country. Parents with inclusion criteria were identified by a trained expert in each hospital. The objectives of the study were stated for the parents. If the parents met the inclusion criteria, they were asked to complete and sign the informed consent form to participate in the study. After obtaining written consent, the Parental Stress Questionnaire, the General Health Questionnaire, were completed by an expert through interviews with parents and by interviewing mothers after 24 h of infants' hospitalization in the NICU.

**Fig. 1:**
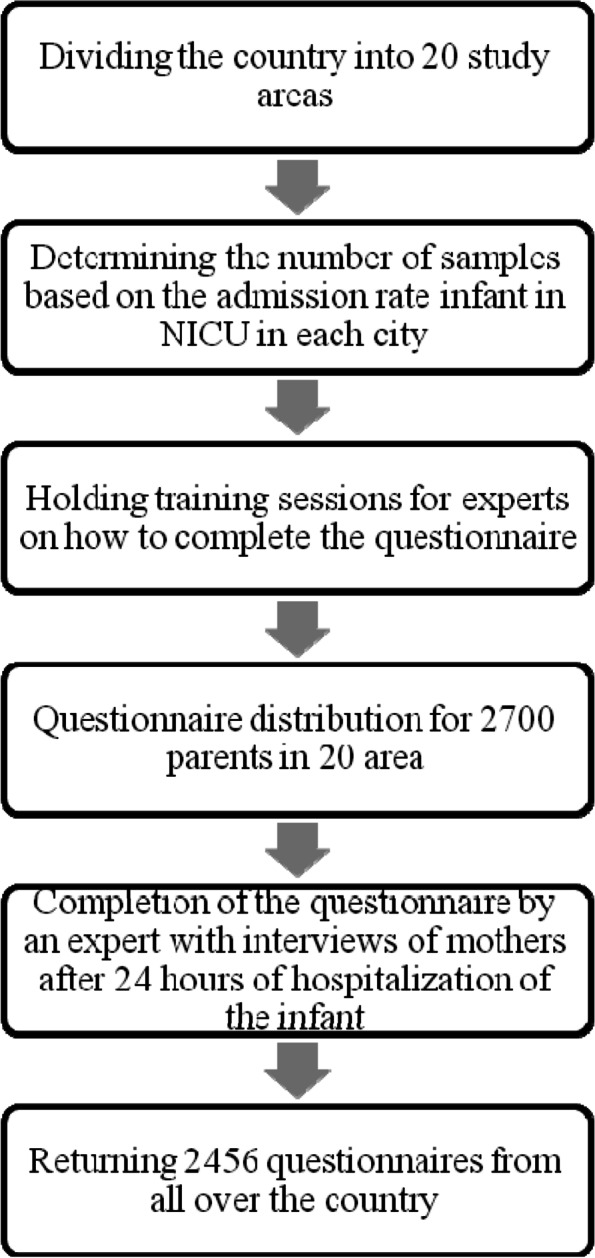
Steps of study

Two thousand seven hundred questionnaires were distributed and 2456 questionnaires were received. Missing data was < 5% percent. The sampling method was multi-stage random (more than one method). First, the whole country was divided into 10 health zones of the Ministry of Health. In the next stage, the Stratified sampling was used. Our stratums were in the health zone. The number of hospital beds was considered as the sampling weight; statistics were extracted from the Health Department of the Ministry of Health and Medical Education. The number of samples in each hospital was selected in proportion to the number of NICU beds. After that, in each hospital, sampling was done by census manner of parents of infants admitted to the NICU who met the inclusion criteria, with informed consent. The sampling period lasted 8 months. Inclusion criteria included parents with infants hospitalized in the NICU, 24 h after hospitalization of the infant, willingness to participate in the study. Exclusion criteria included unwillingness to continue participating in the study (Fig. 1).

#### Parental stress questionnaire

The Parenting Stress Questionnaire had 49 questions or items that were organized into two parts: the first part was related to demographic information of parent and child contexts with 15 questions, and the second part was 26 items related to parental stress questionnaire which included three factors. The first was the measurement of parental stress with 11 items, and the range of factors that aggravated parental stress was the second factor of stress with 13 items, and the factors that relieve parental stress was the third factor with two items. Those with a score of 16.1% had no problem, with grades ranging from 16.2 to 33.1 mild stresses, scores between 33.2 and 50.1 moderate stress, and scores above 50.2 severe stresses.

Validity and reliability of the Parental Stress Questionnaire were conducted by Heidari in 2015 in Iran, with a validity of 78% and reliability of 0.90 [25].

B-28-item general health questionnaire (GHQ): The Goldberg scale is known as the general health or mental health scale and has been used in many studies to determine the level of mental health [26, 27]. Out of 28 items of the questionnaire, items 1 to 7 are related to the scale of physical symptoms. Items 8 to 14 examine the symptoms of anxiety and sleep disorders, and items 15 to 21 are related to the assessment of social functioning symptoms, and finally, items 22 to 28 assess the symptoms of depression. GHQ questionnaire are scored on the Likert scale from zero to three and a lower score shows a better mental condition. Through the sum of items, the scores for the subscales are 0–21 and the scores of the total scale are 0–84. The validity of the general health questionnaire was reported to be 87% and its reliability was reported to be 0.89 [28].

### Data analysis

To determine the parental mental status and to investigate the relationship between the effective factors, the data were analyzed with descriptive and analytical statistics using SPSS 25. In this way, first, the normality of the variables was determined using the Kolmogorov–Smirnov scientific test. Then parametric tests were used, such as one-way analysis of variance, independent t-test, and chi-square, and Spearman's correlation coefficient test. Independent t-test was used to assess stress level in fathers and mothers. Chi-square test was used to determine the difference between parents' stress according classification of stress score. One way test was used to determine the difference in the length of hospital stay in terms of different levels of parental stress scores. Spearman's correlation was used to determine the relationship between the length of hospitalization and parental stress in NICU.

## Result

### Participants

44.9% of the samples were fathers and 55.1% of the samples were mothers. The majority of parents had a diploma (38.3%). Most of the women (44.3%) were homemakers. A large proportion parents lived in area 10 (17.2%). The majority of parents (89%) had no history of infertility. Regarding the type of pregnancy (wanted or unwanted), the majority of infants admitted to the intensive care unit were wanted (82.2%). More than half of parents (62%) were Persian. The majority of parents (67.8%) lived in the city. Just over half of infants (55.7%) were boys. The infant's previous hospitalization history in the NICU was 68.8%. The highest cause of hospitalization was due to prematurity with 49.1%. The majority of infants (60.5%) were the first child in the family. The average age of parents was 31.59 years with a standard deviation of 6.6 years. The mean age of hospitalized neonates was 35.7 days with a standard deviation of 180.9 days. The mean weight of hospitalized neonates was 2372.1 g with a standard deviation of 895.9 g. The mean height of hospitalized neonates was 45.7 cm with a standard deviation of 6.3 cm. The mean duration of maternal infertility before pregnancy was 6.001 years with a standard deviation of 4.16 years. The mean hospital stay of hospitalized neonates was 11.5 days with a standard deviation of 13.1 days. The demographic factor of ethnicity is one of the factors affecting the level of stress. Baloch ethnicity with a mean score of 11.52 had the highest level of stress.

### Stress level and mental health in parents

The mean score of mothers in the factor of stress was higher. Independent t-test showed a statistically significant difference between parents in the amount of stress for the level of ten percent, respectively (Table1).

**Table 1:** Stress level in fathers and mothers with infants admitted to the NICU

Variable	Stress level	
Parents	**Mean**	**(SD)**
Father	42.97	12.98
Mother	48.08	13.19
Independent t-test	-9.606	
*P* value	*p < .001	

According to Table 2, the Chi-square test showed a statistically significant difference between father, mother, and the amount of stress. The level of stress in mothers was significantly higher than in fathers (*p* < 0.001).

**Table 2:** Classification of stress score based on the parental type of parents with infants admitted to the NICU

**Stress level**	0–16.1	16.2–33.1	33.2–50.1	< 50.2	Total
**Variable**					
**Father**	17	275	447	351	1090
**Mother**	19	197	487	663	1366
**Total**	36	472	934	1014	2456
**Chi square**	80.717				
***P* value**	*p < .001				

Table 3 showed that the mean score of all dimensions of physical symptoms, anxiety, social functioning, depression, and total mental health score in mothers was higher than fathers.

**Table 3:** Comparison of mean scores of physical symptoms, anxiety, social functioning, depression and total mental health score in fathers and mothers

	physical		anxiety		social		depression		**Mental health**	
Variable	**Mean**	**(SD)**	**Mean**	**(SD)**	**Mean**	**(SD)**	**Mean**	**(SD)**	**Mean**	**(SD)**
Father	12.73	3.41	13.65	4.21	15.62	3.81	9.74	3.74	51.35	10.43
Mother	14.40	3.96	15.47	5.22	15.30	3.71	10.38	4.39	55.56	12.59
Independent t-test	-11.414		-9.723		2.060		-3.950		-8.351	
*P* value	*p < .001		*p < .001		*p = .040		*p < .001		*p < .001	

The results revealed that the variables of parents' age, infertility time, child age, height, and weight had no statistically significant relationship with stress. The results demonstrated no statistically significant relationship between different levels of education and stress levels. The results also revealed there was no statistically significant relationship between parents' jobs and stress levels. No significant difference between parents' place of residence (urban and rural) and their stress scores was observed. There is no significant relationship between the stress score of parents and the sex of their infants. Besides, no significant relationship between parents' stress scores and their child's hospitalization history was seen. There is no significant difference between the cause of the infant's hospitalization and their parents' stress scores. There was no significant difference in parental stress according to the variable of childbirth rank.

Table 4 showed that there was a statistically significant difference in the length of hospital stay in terms of different levels of parental stress scores. It can be said that with increased hospitalization time, the amount of parental stress increased. Spearman's correlation was used to determine the relationship between the length of hospitalization and parental stress in NICU. There was a strong and positive correlation between the length of hospitalization and stress, which was statistically significant (Table 5).

**Table 4:** Classification of stress scores based on the length of hospitalization of the infant in the NICU

Variable	length of hospitalization		
Stress level	**N**	**Mean**	**(SD)**
0–16.1	27	41.8	91.8
16.2- 33.1	394	17.10	21.12
33.2–50.1	768	91.10	53.12
50.1 <	847	78.12	11.14
ANOVA	04.5		
*P* value	*p = .002

**Table 5:** Relationship between the length of hospitalization and parental stress in NICU

**Variables**	r_s_	Critical value	Level of significance
Parental stress	0.123^*^	*p < .001	0.01
Length of hospitalization			

^*^Correlation is significant at the 0.01 level (2-tailed)

Table 6 showed that the percentage score of each question showed item 10: In this situation, I pray to God for help" 12.8 percent and item 11: "In this situation, I expect the medical staff to give me a definite answer about the baby's health." 8.1%, was the highest percentage.

**Table 6:** Stress level in parent (neonatal parents questionnaire)

QUESTION	I have not experienced it at all		very little		Little		Medium		much		Very much		I have fully experienced	
	N	%	N	%	N	%	N	%	N	%	N	%	N	%
I have felt stressed ever since the baby was admitted to the hospital	254	7/8	355	2/12	248	5/8	553	19	689	7/23	647	3/22	157	4/5
I feel worried because my baby is in the hospital	109	7/3	276	4/9	252	6/8	491	8/16	835	5/28	859	3/29	105	6/3
I feel worried because my baby may die	471	5/16	480	8/16	292	2/10	382	4/13	498	4/17	647	6/22	90	1/3
I feel uncomfortable in the environment of the hospital	535	7/18	534	7/18	406	2/14	505	7/17	415	5/14	388	6/13	76	7/2
I have become oversensitive ever since the baby was admitted to the hospital	440	3/15	463	1/16	388	5/13	499	4/17	528	4/18	484	8/16	72	5/2
I can hardly eat or sleep after the baby was admitted to the hospital	351	3/12	463	2/16	364	7/12	626	9/21	510	8/17	452	8/15	97	4/3
My everyday life has been disrupted	295	4/10	366	9/12	295	4/10	522	4/18	601	1/21	633	3/22	131	6/4
I have grown exhausted from the period of time the baby has stayed in the hospital	230	9/7	402	8/13	336	6/11	527	2/18	635	9/21	656	6/22	117	4
I feel depressed by the period of time the baby has stayed in the hospital	510	7/17	485	8/16	368	8/12	505	5/17	448	6/15	475	5/16	90	1/3
In this situation, I pray to God for help	78	7/2	117	4	100	5/3	288	9/9	598	6/20	1347	5/46	370	8/12
In this situation, I expect the medical staff to give me a definite answer about the baby’s health	128	4/4	135	7/4	111	8/3	318	11	631	9/21	1328	46	235	1/8

## Discussion

Our study is the first national study with a large number of samples to determine parental stress. We found that mothers had more stress than fathers. The results of our study were not confirmed by the study of Noergaard et al. [18]. On the other hand one study in India showed parents had the same stress when the infant was hospitalized in the NICU [21]. But other quantitative studies confirmed our results [19, 20]. As stated in various studies [14, 24], these differences are due to differences in care conditions. What is important is that according to the results of this study, mothers of hospitalized infant in NICU in Iran need screening services and appropriate mental support actions. Therefore, psychological counseling of parents, especially mothers, should be a priority for health policymakers. A researcher stated, to reduce parents' stress and improve their health, appropriate interventions are recommended, such as empowering parents in the NICU [29]. A researcher stated that mothers experience a lot of stress in the NICU and to provide holistic care, both infants and parents should be considered [17].

As the previous study recommended, in order to reduce stress, it is very important to know the factors related to stress [22, 23, 30]. According to the results of a study, nurses should identify sources of stress in parents so that they can guide and support them properly [31]. Our results showed that the factors affecting parental stress include location, parental ethnicity, and length of hospitalization. The length of hospitalization of the infant was one of the factors related to stress. In our study, the stress of the parents increased with the length of hospitalization, and other studies also confirmed this finding [21]. Meanwhile, another study stated the mother's low education level and VLBW infants as factors related to increased stress [14]. Since parental stress is a global issue in NICU [15], we suggest that health policymakers should pay attention to ethnic differences to provide appropriate interventions. In addition, parents of infants with longer hospitalization should be considered high-risk parents. The results of another study showed that it is essential that caregivers recognize high-risk mothers and help them recover as soon as possible by starting interventions [32]. Based on our results, the longer the hospital stay, the more stressed the parents become. It is recommended that these parents be considered as high-risk parents, and proper ward design and the existence of appropriate conditions in ward design can enhance parental involvement. The results of a study showed that maternal stress has negative results in breastfeeding and considering the benefits of breastfeeding, it is necessary to evaluate the stress and plan appropriate interventions [33].

The results of our study showed that the majority of parents experienced moderate to severe stress and mental health scores. Lack of decisive response to parents was one of the most stressful issues. Several study showed a high level of parental stress [7, 21, 34]. Meanwhile, another study reported a lower level of stress due to the presence of supportive factors such as family [14]. One researcher stated that NICU culture needs to change towards parental psychological support [35]. The results of another study showed that parents felt anxious because of invasive interventions and complex intensive care technology, which harms parents' quality of life [36]. According to the results of a systematic review, since the premature baby births have increased and the NICU is the extra-uterine place for newborns, paying attention to the proper design of this unit can promote the health of the infants and parents and prevent adverse consequences [37]. We suggest that, to reduce stress and improve the mental health of parents, neonatal health planners should take appropriate interventions to screen high-risk parents and then psychological counseling be recommended by a professional nurse. The researcher said that policymakers should pay more attention to parents' social support such as providing accommodation, easy access to the hospital, and assistance with their transportation services. Improving such policies promotes maternal and infant health [38]. One study showed that support programs are necessary to prevent psychological trauma in mothers with hospitalized infants and also to develop an appropriate program to identify the factors affecting stress [39].

## Conclusion

This study shows that parents with infants admitted to the NICU experience moderate to severe stress. In addition, mothers' stress was higher than fathers'. It also shows that ethnicity, place of residence, and length of hospitalization of the infant are among the factors affecting parental stress. Also, based on our results, the Parental Stress Questionnaire can be used as a stress screening tool in the neonatal intensive care unit. So that the care team can make appropriate interventions for the parents based on the screening results. The results of our study can be used to develop future studies such as the design of intervention to reduce parental stress according parental stress screening especially for high-risk mothers. Also, the health policy maker should pay attention to stress risk factors in NICU.

### What is already known on this topic

- More attention is paid to the survival and treatment of the sick infant in the neonatal intensive care unit and parental stress is not considered.
- The results of studies on parental stress vary in the neonatal intensive care unit

### What this study adds

- Parents experience moderate to severe stress in the NICU. Mothers' stress was higher than fathers then mothers are at higher risk for mental health disorders.
- Health policymakers should pay attention to parental stress and stress risk factors to provide appropriate and immediate interventions in the NICU.
- Ethnicity, place of residence, and length of hospitalization of the infant are among the factors affecting parental stress

## Data Availability

Data were generated at Shahrekord University of Medical Sciences. Derived data supporting the findings of this study are available from the corresponding author [Haydeh Heidari] on request.

## Abbreviation

NICU: Neonatal intensive care unit
